# Melatonin inhibits senescence-associated melanin pigmentation through the p53-TYR pathway in human primary melanocytes and the skin of C57BL/6 J mice after UVB irradiation

**DOI:** 10.1007/s00109-023-02301-y

**Published:** 2023-04-10

**Authors:** Li-Ping Ma, Meng-Meng Liu, Fang Liu, Bo Sun, Si-Nian Wang, Jie Chen, Hui-Jie Yu, Juan Yan, Mei Tian, Ling Gao, Qing-Jie Liu

**Affiliations:** 1 China CDC Key Laboratory of Radiological Protection and Nuclear Emergency, National Institute for Radiological Protection, Chinese Center for Disease Control and Prevention, 100088 Beijing, China; 2 Department of Dermatology, Chaoyang Hospital, Capital Medical University, 100020 Beijing, China; 3 PLA Rocket Force Characteristic Medical Center, 100088 Beijing, China

**Keywords:** Ultraviolet B, Melanocytes, Melanin, Premature senescence, p53, TYR

## Abstract

**Abstract:**

UVB exposure accelerates skin aging and pigmentation. Melatonin effectively regulates tyrosinase (TYR) activity and aging. The purpose of this study was to determine the association between premature senescence and pigmentation, and the mechanism of melanin synthesis effected by melatonin. Primary melanocytes were extracted and identified from the male foreskin. To inhibit TYR expression, primary melanocytes were transduced with the lentivirus pLKD-CMV-EGFP-2A-Puro-U6-TYR. The wild-type *TYR*^*(*+*/*+*)*^ and *TYR*^*(–/–)*^ or *TYR*^*(*+*/–)*^ knockout C57BL/6 J mice were used to determine the role of TYR on melanin synthesis in vivo. Results showed that UVB-induced melanin synthesis is dependent on TYR in primary melanocytes and mice. Furthermore, in primary melanocytes pretreated with Nutlin-3 or PFT-α to up or downregulate p53, results showed that premature senescence and melanin synthesis increased in primary melanocytes after UVB irradiation at 80 mJ/cm^2^, and further increased after being treated with Nutlin-3, while significantly decreased with PFT-α. In addition, melatonin inhibited UVB-induced premature senescence associated with inactivation of p53 and phosphorylation of p53 on Ser15 (ser-15), a decrease of melanin synthesis accompanied by reduced TYR expression. Moreover, skin erythema and pigmentation induced by UVB were reduced in the dorsal and ear skin of mice topically pretreated with 2.5% melatonin. These indicate that melatonin inhibits UVB-induced senescence-associated pigmentation via the p53-TYR pathway in primary melanocytes and prevents pigmentation obviously in the dorsal and ear skin of C57BL/6 J mice after UVB irradiation.

**Key messages:**

- P53 links UVB irradiation-induced senescence and senescence-associated pigmentation and regulates TYR in primary melanocytes after UVB irradiation.
- Melatonin inhibits senescence-associated pigmentation through the p53-TYR pathway in primary melanocytes.
- Melatonin prevents skin erythema and melanin pigmentation induced by UVB irradiation in the dorsal and ear skin of C57BL/6J mice.

**Supplementary Information:**

The online version contains supplementary material available at 10.1007/s00109-023-02301-y.

## Introduction

The skin is a physical barrier that protects the inner parts of the body from the surrounding physical, chemical, and biological environments and helps to resist adverse agents [1]. The human skin is repeatedly exposed to ultraviolet (UV) irradiation, with affects skin cell survival and activity and leads to skin damage, inflammation, aging, and skin pigmentation [2]. Furthermore, skin exposed to UV irradiation can affect the overall stability of individuals by activating the central neuroendocrine system [3]. Melanin is a skin pigment that as the first line of defense blocks UV irradiation and dissipates UV as heat preventing any harm. It is generated in melanin-producing cells known as melanocytes [4]. Melanocytes mainly exist at the epidermal and dermal junction, accounting for about 10% of basal epidermal cells, and producing protective melanin [5]. Tyrosine is the starting material for melanin production, and tyrosinase is used for converting precursor tyrosine to DOPA and, subsequently, melanin [6]. UVB can penetrate the epidermis, cause DNA damage, and promote the accumulation of reactive oxygen species (ROS) in the melanocytes [7]. Melanocytes coordinate the repair of UVB-induced DNA damage by blocking the cell cycle. Cell division stops, and premature senescence (PS) was induced when repairs are ineffective or DNA damage persists [8]. Premature senescence has specific physiological alterations, such as reduced cell proliferation, larger cell volume, a higher lysosome quantity of lysosomes, and senescence-associated gene upregulation. PS can be triggered by several factors, such as oxidative stress and DNA damage [9]. As a tumor suppressor, p53 maintains the intact genome by modulating cell apoptosis and arresting growth during DNA damage response. Upon irradiation, the mutant gene products within ataxia telangiectasia and ATM stabilizes and activates the p53 through phosphorylation in positions ser-15, ser-20, and ser-46 [10, 11]. The phosphorylation of p53 in positions ser-15 and ser-20 can enhance p53 accumulation and activate DNA repair. However, the phosphorylation of p53 in position ser-46 tightly regulates cell apoptosis post-DNA damage [12]. Kang identified three significant pathways regulating senescence. The p16 and p53-mediated pathways induced PS by regulating the cell cycle [13].

Melatonin, an indolic hormone, can be produced from the pineal gland, synthesized in the human skin, and has several functions [14, 15]. Melatonin and its metabolites limit oxidative stress by scavenging toxic ROS, inhibiting ROS generation, and stimulating the production of antioxidant enzymes [16, 17]. Additionally, melatonin also has anti-inflammatory [18] and anti-apoptotic effects [19, 20]. Melatonin and its corresponding metabolites can regulate the skin and facilitate the development of potent anti-aging molecules [21]. They also strongly influence melatonin production and regulate tyrosinase activity [22]. Therefore, we determined the key factors of senescence-associated pigmentation and the underlying mechanism in premature senescence and senescence-associated pigmentation in the melanocytes after UVB irradiation.

## Materials and methods

### Cell culture

Human immortalized keratinocytes (HaCaT) were purchased from the Cell Resource Center of the Institute of Basic Medical Sciences, Peking Union Medical College Hospital (IBMS, CAMS/PUMC). The primary melanocytes (MC) were cultured from adult healthy male foreskin tissues in our laboratory, and the second to fourth generations of cultured primary melanocytes were used in subsequent experiments.

Primary melanocytes were acquired from five men who had routine circumcisions performed at Chaoyang Hospital and the PLA Rocket Force Characteristic Medical Center in Beijing, China. Adult male foreskin samples were disinfected with 70% ethanol for disinfection and washed with phosphate-buffered saline (PBS) solution. After the fat and subcutaneous tissue were removed, the prepuce tissue was cut into 3 mm wide strips. After digesting the prepuce in 4 °C refrigerators for 18 h with 0.25% trypsin solution, the epidermis and dermis were separated with a blade. Primary melanocytes were isolated from the prepuce’s middle layer and cultured in the M254-medium (Gibco, USA) with 1% penicillin–streptomycin (Beyotime Biotechnology, Shanghai, China) and 1% human melanocyte growth supplement-2 (HMGS-2, Gibco, USA) at 5% CO_2_ and 37 °C. HMGS-2 contains essential substances for melanocyte growth but inhibits keratinocytes and fibroblasts. Keratinocytes and fibroblasts were gradually removed from the wall after replacing the culture medium 2–3 times. Similarly, the HaCaT cells were cultivated in the MEM/EBSS (Hyclone, South Logan, UT, USA) containing 1% penicillin–streptomycin and 10% fetal bovine serum (FBS, Hyclone, South Logan, UT, USA) at 5% CO_2_ and 37 °C.

### Animals

The 8-week-old *TYR*^*(–/–)*^ and *TYR*^*(*+*/–)*^ knockout C57BL/6 J mice were obtained from the GemPharmatech Company (Jiangsu, China) and the age-matched wild-type *TYR*^*(*+*/*+*)*^ C57BL/6 J mice from the Charles River Laboratory Animal Center (Beijing, China). The Experimental Animal Welfare Committee of the National Institute for Radiological Protection (NIRP, act no. 2021–009) of the Chinese Center for Disease Control and Prevention (China CDC) approved our animal experiment protocols. The study was conducted following the Chinese regulations for animal experimentation (Ministry of Agriculture, act no. 2001–464, 29 May 2001). We used wild-type *TYR*^*(*+*/*+*)*^, *TYR*^*(–/–)*^*,* and *TYR*^*(*+*/–)*^ C57BL/6 J mice, three of each type, to explore the effects of TYR on melanin synthesis. In addition, 12 wild-type *TYR*^*(*+*/*+*)*^ C57BL/6 J mice were randomized into non-treated, UVB, UVB + Vaseline, and UVB + 2.5% MT groups, 3 animals per group (*n* = 3), to determine the role of melatonin in UVB-induced skin morphological changes and melanin synthesis.

### UVB irradiation

Logarithmic cells incubated with 2 mL of PBS were irradiated with 80 mJ/cm^2^ of UVB for 46 s at 1.5 mW/cm^2^ power density using a UVB lamp (311–313 nm) (model: SH4B-T UV, SIGMA, Shanghai, China). The dose was calibrated before irradiation using a TN-2340 ultraviolet intensity meter, and the correction coefficient value was 1.16. The light source was placed approximately 40 cm away from the cell. A uniform (1%) homogenous field of 10 cm × 15 cm was prepared for UVB irradiation. Cells were cultivated at 5% CO_2_ and 37 °C.

The mice were anesthetized and depilated to expose about 3 cm × 5 cm of the skin on the back. After 30 min of pre-treatment with melatonin, the exposed skin on the back and ear was irradiated with a 600 mJ/cm^2^ dose of UVB for 130 s at 4 mW/cm^2^ power density using a UVB lamp (311–313 nm), and the changes in the skin on the back and ear were observed at 0, 48, and 96 h after irradiation. A TN-2340 ultraviolet intensity meter was used to calibrate the dose before irradiation, and the correction coefficient value was 1.16. The light source was about 15 cm away from the skin on the back.

### Preparation and treatment of melatonin formulations

The Sigma-Aldrich Company (Merck, Darmstadt, Germany) supplied melatonin powder. After completely dissolving 50 mg of melatonin powder in 0.5 mL absolute ethanol, PBS was added until the total volume of the solution was 2.15 mL to obtain the 0.1 mol/L melatonin storage solution. Diluting the concentrations with PBS yielded the other concentrations. The cells were pretreated with a 10^−5^ mol/ L melatonin solution for 12 h before irradiation. The composition of the melatonin ointment was given in weight per weight (W/W) percentages. First, solution 1 was prepared by mixing 100 µL of anhydrous ethanol and 200 µL of Tween-20 (Solarbio Company, Beijing, China). Then, a 1% melatonin ointment was prepared by adding 1 mg of melatonin powder to solution 1, and Vaseline was added to increase the total weight of the mixture to 1 g. Also, 2.5% and 5% melatonin ointments were prepared using this method and the corresponding required percentage of the individual components. The exposed back and ear skin of mice was evenly and thinly coated with melatonin ointment for 30 min before irradiation.

### shRNA transfection

The pLKD-CMV-EGFP-2A-Puro-U6-TYR lentivirus vector was purchased from OBIO Technology (Shanghai, China) and transfected into the primary melanocytes at MOI = 40 using pLKD-CMV-EGFP-2A-Puro-U6-NC as the negative control (NC). A fluorescent microscope was used to detect the GFP protein level at 200 × and determine the lentivirus transfection efficiency (Thermo Scientific, Waltham, MA, USA) after 24 h. Then, the M-PER® Mammalian Protein Extraction Reagent (Thermo Scientific, Waltham, MA, USA) was used to extract the total protein in the subsequent assays. Three duplications of cell samples per group for each experiment, and this experiment was repeated three times.

### Automated capillary electrophoresis Western blotting analysis

After cell lysis for 30 min at 4 °C using the RIPA buffer (Beyotime Biotechnology, Shanghai, China) containing protease and phosphatase inhibitors (Roche, Basel, Switzerland), the cells were centrifuged at 12,000 r/min using the cell lysates. The supernatant protein content was determined using the Bicinchoninic acid (BCA) kit (Thermo Fisher Scientific, Waltham, MA, USA). After extraction, a 5 × master mix with 0.1 × sample buffer was used to dilute cellular proteins using the relevant experimental kit. Then, the primary antibody was diluted using antibody diluent II provided in the kit. The diluted protein, diluted primary antibody, HRP-labeled secondary antibody, antibody diluent II, washing solution, and luminol-conjugate mix were then poured into each well of the plate provided in the kit. Finally, the proteins were fractionated, immobilized, and immunologically detected using the automatic capillary electrophoresis Western System (ProteinSimple, San Jose, CA, USA). The Compass for SW 4.0 software (ProteinSimple, San Jose, CA, USA) was used to quantify and visualize the proteins. The antibodies used were mouse anti-p53 (1C12) mAb (#2524S, CST, 1 : 50), rabbit anti-phospho-p53 (Ser15) antibody (#9284, CST, 1 : 50), the mouse anti-Tyrosinase antibody (T311) (sc-20035, Santa Cruz Biotechnology, 1 : 10), and mouse anti-β-actin mAb (#3700, CST, 1 : 50). Three duplications of cell samples per group for each experiment, and this experiment was repeated three times.

### Quantitative real-time PCR (qRT-PCR)

The TRIzol reagent (Ambion, Austin, TX, USA) was used to extract the total RNA, which was later used to prepare cDNA using the PrimeScript™ II 1st Strand cDNA Synthesis Kit (TaKaRa, Tokyo, Japan) by reverse transcription. After that, 7500-Fast Real-time PCR (Thermo Company, USA) was used to conduct qRT-PCR. The expression of target genes was determined using the ∆∆CT approach, with β-actin as the reference. The sequences of tyrosinase (TYR) primers were 5′-TTGTGAGCTTGCTGTGTCGT-3′ (forward) and 5′-GTCAGGCTTTTTGGCCCTAC-3′ (reverse).

### DOPA staining

The cells were added to each well with 1 mL of 4% paraformaldehyde (PFA) (Solarbio Company, China) after being washed with PBS. They were then fixed on a shaking table for 15 min. After that, PBS was used to rinse the cells three times for 3 min, and 1 mL of 0.3% TritonX-100 (Sigma Company, USA) was added to the cells per well for 30 min before washing three times with PBS. The staining group cells were mixed with 1 mL of 0.1% concentration L-DOPA solution (Sigma Company, USA) per well, preheated at 37 °C, and incubated for 4 h at 37 °C. The control cells were treated with 1 mL of PBS per well and photographed at 400 × magnification, and the PBS-incubated cells were used as a control. When the cells turned black, this confirmed the formation of melanin. The optical density ratio to total area (IOD/ARE) of the stained black cells was quantified using the Image-Pro Plus software (Media Cybernetics, USA). Three duplications of cell samples per group for this experiment.

### Melanin content assay

Seventy two hours after UVB irradiation, we harvested and rinsed the cells three times with PBS and added 1 mol/L of NaOH. Then, the resultant mixture (100 µL) was added to the 96-well plates, followed by incubation at 37 °C for 60 min. Finally, a microplate reader (Multiskan MK3, Thermo Electron Corporation, MA, USA) was used to measure the absorbance (OD) value at 492 nm (OD492) to determine the melanin content. Three duplications of cell samples per group for this experiment.

### Measurement of tyrosinase activity

Seventy two hours after cells were seeded into 96-well plates and exposed to UVB irradiation, 1% Triton X-100 buffer (100 µL) was added to each well and shaken for 15 min. After that, 0.1% 3, 4-Dihydroxy-L-phenylalanine (L-DOPA) buffer (100) was added to each well, followed by incubation at 37 °C for 2 h. Similarly, we determined the OD492 value to assess tyrosinase activity. Three duplications of cell samples per group for this experiment.

### Analysis of senescence-associated beta-galactosidase (SA-β-gal) activity

The cells were stained following specific instructions using the SA-β-gal staining kit (Beyotime Biotechnology, Shanghai, China). Briefly, after washing with PBS, the cells were fixed using the fixation solution at ambient temperature for 15 min followed by overnight incubation at 37 °C with the staining solution. An optical microscope was used to analyze the results from three randomly selected fields at 200 × magnification and count the number of stained blue cells. Finally, the Image-Pro Plus 6.0 software (Media Cybernetics, Silver Spring, USA) was used to calculate the proportion of SA-β-gal-positive cells. Three duplications of cell samples per group for this experiment.

### Giemsa staining

Seventy two hours after the primary melanocytes were irradiated with 80 mJ/cm^2^ UVB, they were fixed with methanol for 15 min. The primary melanocytes were removed and stained with Giemsa working solution for 15 min and washed with PBS solution. A microscope was used to measure the size of the cells and nuclei. Three duplications of cell samples per group for this experiment.

### Lyso-Tracker Red staining

The primary melanocytes were incubated with Lyso-Tracker Red working solution for 30 min. Then, the Lyso-Tracker Red staining working solution was removed, and a normal cell culture medium was added to every well. The fluorescence intensity was then measured and photographed using a fluorescence microscope and imaging system (Olympus). Three duplications of cell samples per group for this experiment.

### Statistical analysis

The GraphPad Prism 9.0 software (San Diego, CA, USA) was used for statistical analysis and plotting. A one-way ANOVA was used to compare several groups, followed by LSD tests, and a two-tailed Student’s *t*-test was used to compare two groups. The data homogeneity of the data was confirmed based on the variances and the normal distribution. The data were presented as the mean ± SD. All differences among and between groups were considered to be statistically significant at *P* < 0.05.

## Results

### Culture and identification of primary melanocytes

**Fig. 1:**
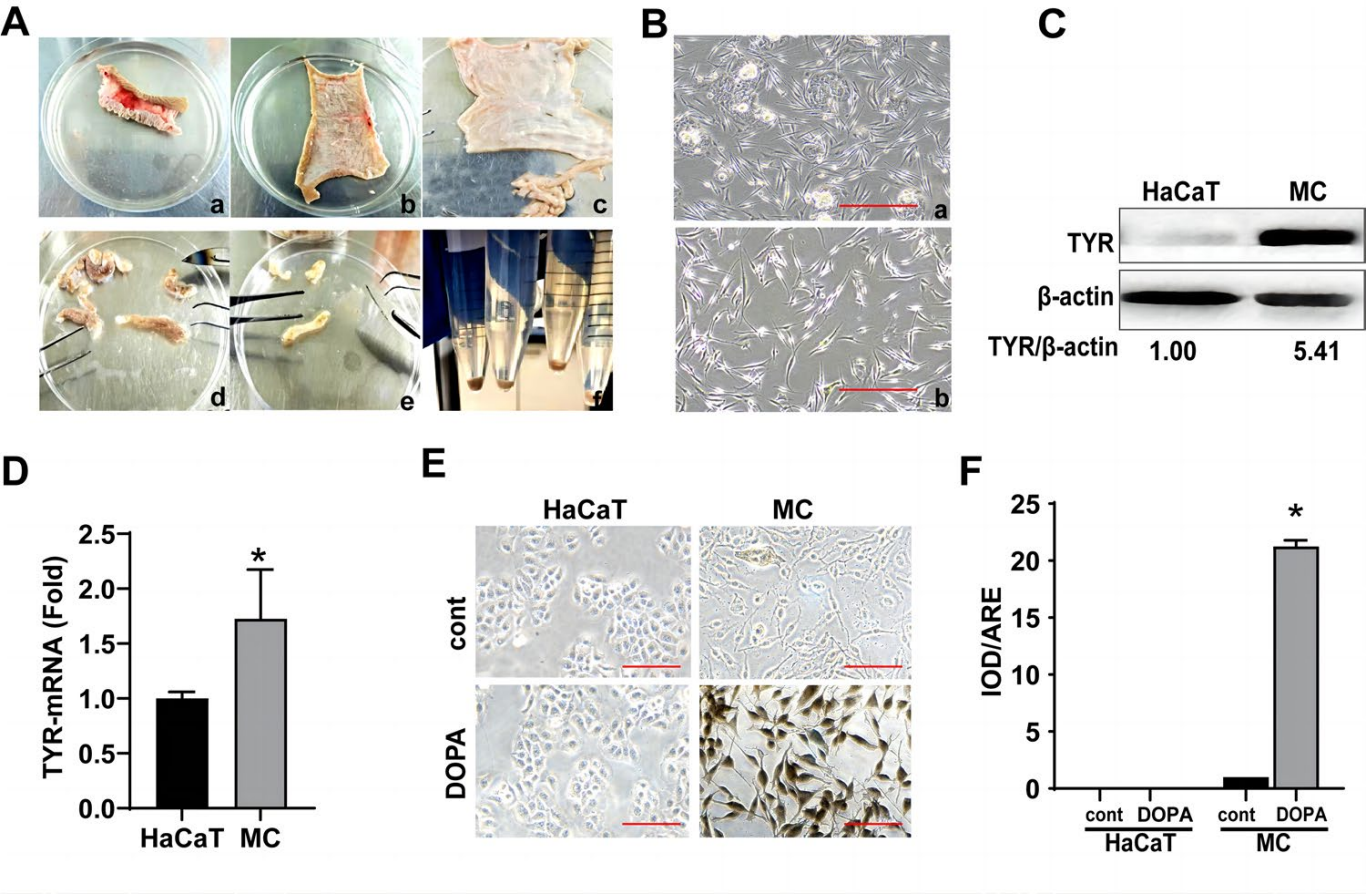
The culture and identification of the primary melanocytes. **A** Steps for primary melanocyte culture. (a) Adult male prepuce tissue was obtained. (b) The fat and subcutaneous tissues were removed from the prepuce. (c) The prepuce tissue was cut into 3 mm strips; (d and e) The epidermis and dermis were separated after 18 h of trypsin digestion; (f) Primary melanocytes were extracted and cultured from the middle layer of the prepuce. **B** Primary melanocytes were observed at 200 × magnification using an optical microscope. (a) Passage 0. (b) Passage 1. **C** and **D** The protein (**C**) and mRNA (**D**) levels of TYR in HaCaT and primary melanocytes. The HaCaT cells were used as control. **E** L-DOPA staining of HaCaT and primary melanocytes. The HaCaT cells were severed as the negative control. **F** Quantification of the optical density ratio to the total area (IOD/ARE) in cells that were stained in black using the Image-Pro Plus software (scale bar indicates 10 µm, *P < 0.05 vs. the control group)

We established primary cultures of adult human melanocytes from the stratum basale of the foreskin after circumcision to better simulate the melanin synthesis process in vivo (Fig. 1A). Passage 0 cells were observed on day 7. They had a spindle shape with a unique vortex arrangement at 90% confluence and were interspersed with keratinocytes and fibroblasts (Fig. 1B(a)). Pure melanocytes were obtained by passaging after trypsin digestion for 2 min (Fig. 1B(b)). Primary melanocytes were identified at the gene, protein, and morphological levels. Primary melanocytes had higher TYR mRNA and protein levels than HaCaT cells (Fig. 1C, D). The primary melanocytes were distinguished by their distinct morphology, dendritic structure, and large cell body. Also, the primary melanocytes appeared black after L-DOPA staining, indicating that the tyrosinase activity was higher in primary melanocytes than that in HaCaT cells (Fig. 1E, F).

### Melanin synthesis is partly dependent on TYR activation and expression in primary melanocytes after UVB irradiation

**Fig. 2:**
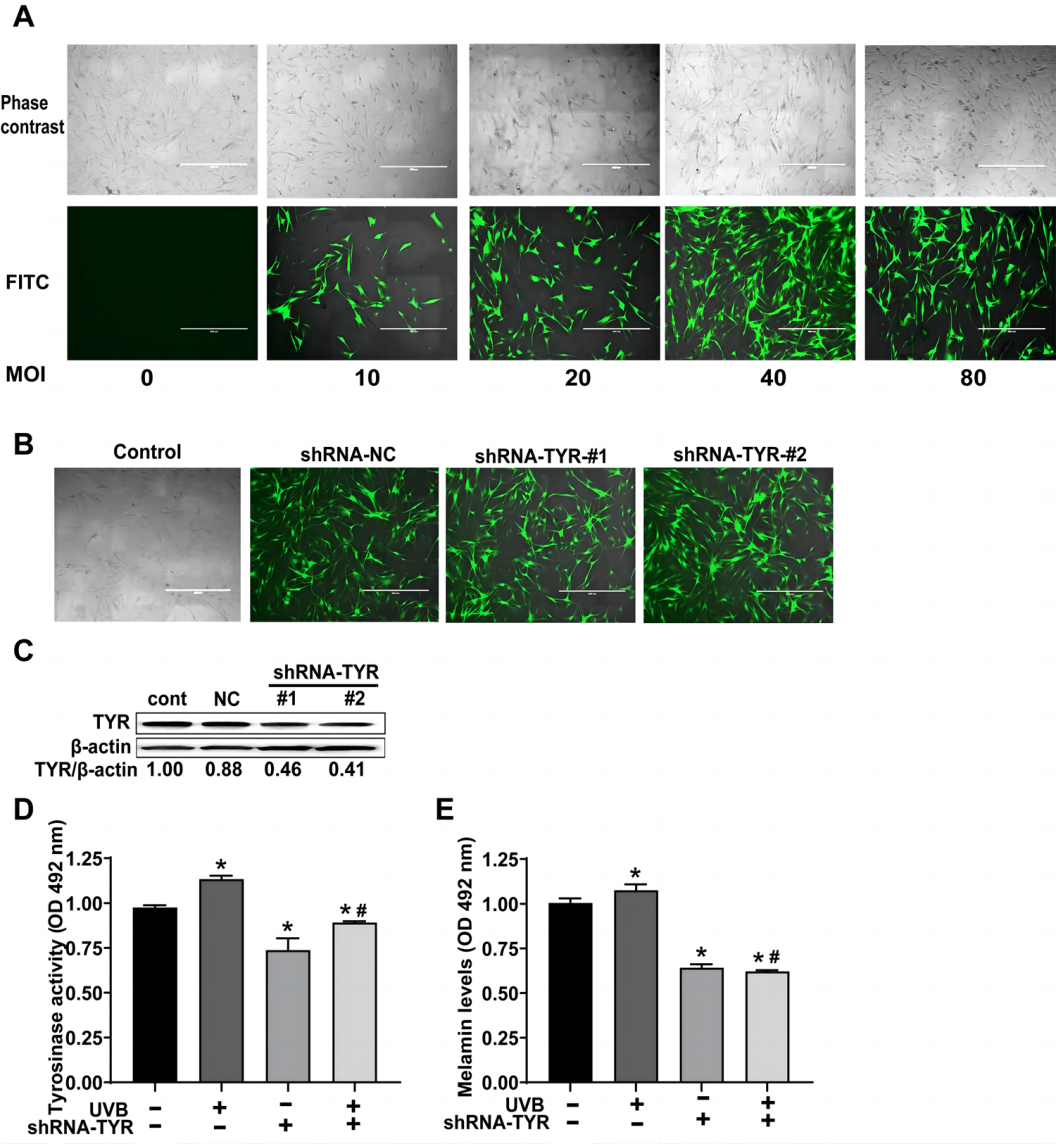
Changes in the tyrosinase activity and melanin level in the TYR-knockdown within primary melanocytes after UVB irradiation. **A** Primary melanocytes transfected with pLKD-CMV-EGFP-2A-Puro-U6-TYR at different multiplicities of infection (MOI). **B** Efficiency of infection in primary melanocytes transfected with pLKD-CMV-EGFP-2A-Puro-U6-TYR at MOI = 40. The images were taken 72 h after infection at a magnification of 200 × . **C** Primary melanocytes were infected with MOI = 40, total proteins were isolated from the cells, and the protein levels of TYR were assessed 72 h after infection. **D** and **E** The tyrosinase activity (**D**) and the melanin levels (**E**) in the TYR-knockdown of primary melanocytes increased 72 h after UVB irradiation. (Scale bar indicates 50 µm, *P < 0.05 vs. the control group; ^#^ *P* < 0.05 vs. the UVB group)

At various multiplicities of infection (MOI), primary melanocytes were transduced with the pLKD-CMV-EGFP-2A-Puro-U6-TYR lentivirus vector. When the MOI was 40, transfected cells showed the most EGFP expression (Fig. 2A, B). The expression of TYR decreased significantly in lentivirus-transduced cells and by nearly 60% in cells transfected with the pLKD-CMV-EGFP-2A-Puro-U6-TYR #2 sequence (Fig. 2C). Furthermore, we discovered that after 72 h of UVB irradiation, tyrosinase activity and melanin levels increased significantly (*P* < 0.05). However, this increase could be alleviated in primary melanocytes by adding pLKD-CMV-EGFP-2A-Puro-U6-TYR #2 transfection (Fig. 2D, E). These findings suggested that UVB-induced melanin synthesis might partly depend on the expression and activation of TYR in primary melanocytes.

### P53 is the key factor linking premature senescence and senescence-associated pigmentation in primary melanocytes after UVB irradiation

**Fig. 3:**
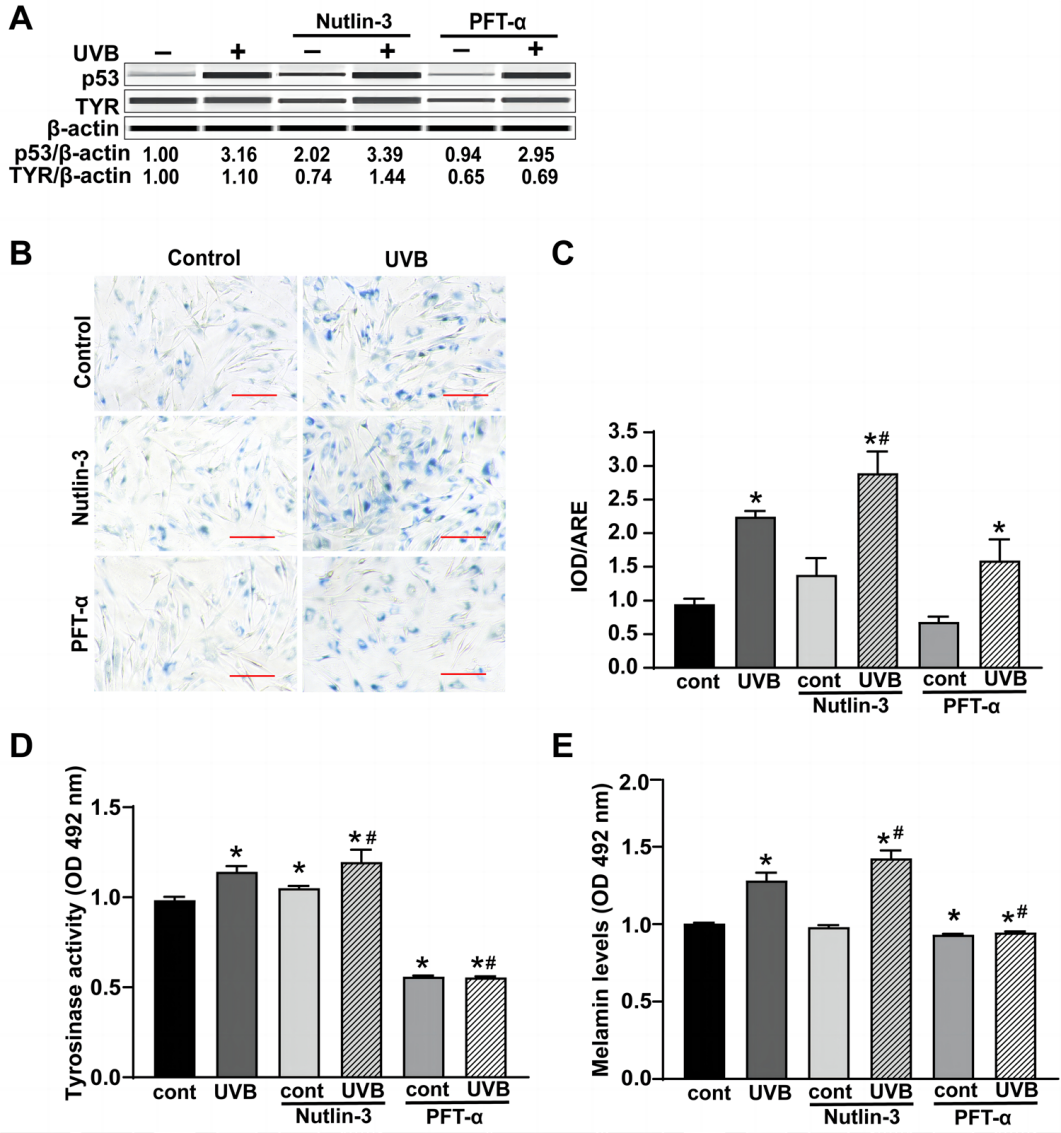
Effects of p53 regulation on premature senescence, TYR expression, tyrosinase activity, and melanin levels in primary melanocytes after UVB irradiation. **A** Changes in the expression of p53 and TYR. **B** Typical SA-β-gal staining images were obtained at a magnification of 200 × . **C** SA-β-gal-positive cell proportion measurement using IPP. **D** and **E** Tyrosinase activity (**D**) and melanin levels (**E**) in primary melanocytes pretreated with 10^–9^ mol/L of Nutlin-3 or PFT-α before UVB irradiation were analyzed 72 h after UVB irradiation. (Scale bar indicates 10 µm, *P < 0.05 vs. the control group; ^#^ *P* < 0.05 vs. the UVB group)

Based on our previous study, UVB irradiation can induce premature senescence of the primary melanocytes and increase p53 expression. The primary melanocytes were pretreated with 10^−9^ mol/L p53 activator (Nutlin-3) and p53 inhibitor (PFT-α) for 12 h before UVB irradiation to upregulate or downregulate the p53 level and to determine the relationship between p53 and TYR (Fig. 3A). The p53 level increased significantly after Nutlin-3 treatment and decreased after PFT-α treatment (Fig. 3A). UVB increased the level of p53 and TYR in primary melanocytes at 72 h after UVB irradiation (Fig. 3A). An increase in p53 and TYR expression induced by UVB irradiation was further enhanced by 8% and 4% after Nutlin-3 treatment, but decreased by 8% and 37%, respectively, after PFT-α treatment (Fig. 3A). UVB irradiation significantly promoted premature senescence, tyrosinase activity, and melanin expression in primary melanocytes (*P* < 0.05). The UVB irradiation-induced premature senescence, tyrosinase activity, and melanin levels increased significantly following the Nutlin-3 treatment. However, they decreased significantly after treatment with PFT-α (*P* < 0.05) (Fig. 3B–E). These results indicated that activation or inhibition of p53 during senescence can strongly influence melanin pigmentation in primary melanocytes after UVB irradiation.

### Melatonin partly inhibits UVB irradiation-induced premature senescence, accompanied by a decrease in p53 levels and phosphorylation of p53 (Ser-15) in the primary melanocytes

**Fig. 4:**
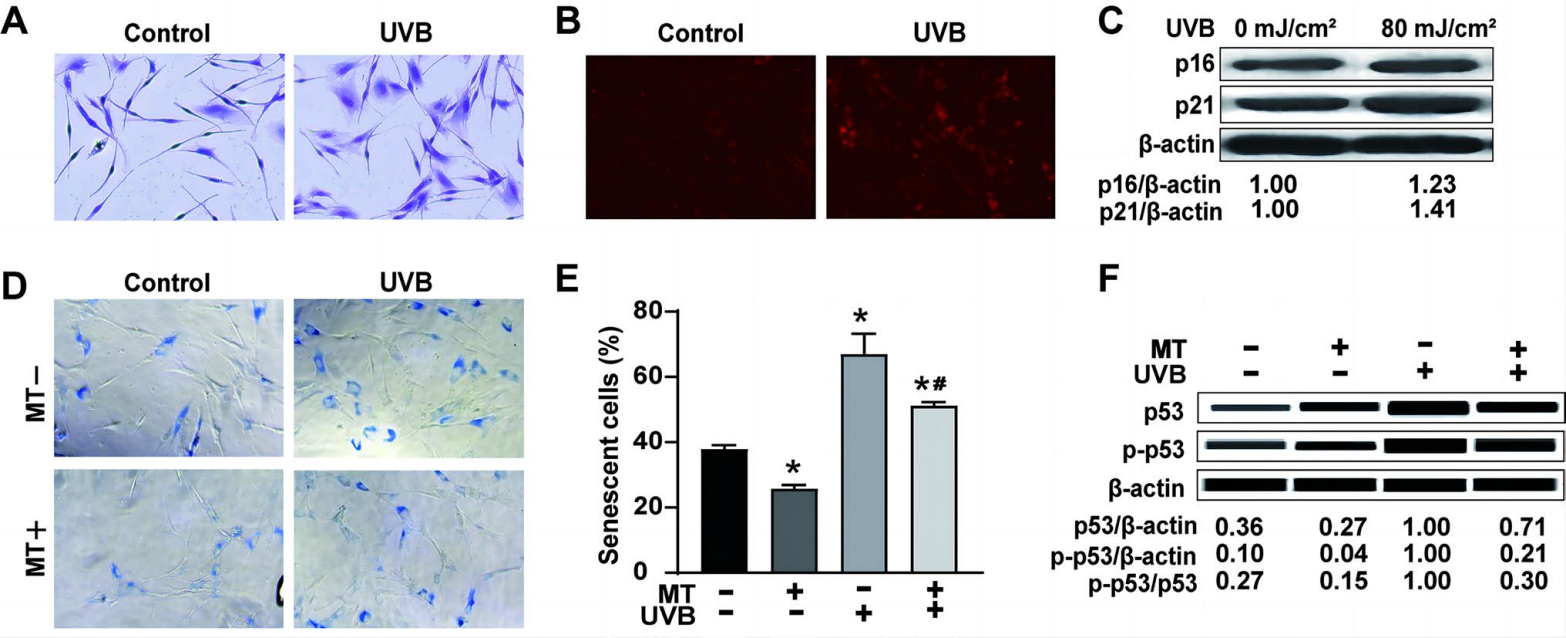
Effects of melatonin on premature senescence, expression of p53 and p-p53 (Ser-15) in primary melanocytes 72 h after UVB irradiation. **A** Morphological changes in melanocytes were observed after UVB irradiation. **B** The representative images of Lyso-Tracker staining were taken after UVB irradiation. **C** The changes in the expression of p16 and p21 protein levels in primary melanocytes after UVB irradiation (80 mJ/cm^2^). **D** The representative images of SA-β-gal staining were taken after treatment with 10^–5^ mol/L of melatonin following UVB irradiation. **E** The proportion of SA-β-gal-positive cells quantified by IPP. **F** The changes in the expression of p53 and p-p53 (Ser-15) in primary melanocytes pretreated with 10^–5^ mol/L of melatonin at 72 h after UVB irradiation (Scale bar indicates 10 µm; *P < 0.05 vs. the control group; ^#^ *P* < 0.05 vs. the UVB group)

To determine the mechanism of melatonin undergoing UVB irradiation-induced premature senescence in primary melanocytes, we irradiated melatonin-treated or untreated primary melanocytes with UVB and detected the proportion of premature senescence and p53 and p-p53 (ser-15) expression at 72 h after UVB irradiation. The cell morphology of primary melanocytes became more prominent, and the cell volume increased at 72 h after UVB irradiation. The results of Giemsa staining showed that the cell morphology changed (Fig. 4A). Lyso-Tracker Red was used to label lysosomes in cells. UVB irradiation increased the number of lysosomes in primary melanocytes (Fig. 4B). UVB irradiation significantly increased the proportion of SA-β-gal-positive cells that stained with blue (*P* < 0.05). It also increased the expression of the senescence-related proteins p21 and p16 (Fig. 4C–E). The results indicated that premature senescence increased after UVB irradiation. However, this increase was partly alleviated after treatment with melatonin (*P* < 0.05). Additionally, UVB irradiation significantly increased p53 and p-p53 levels and the p-p53/p53 ratio (*P* < 0.05). However, these enhancements were inhibited by the additional melatonin treatment (*P* < 0.05) (Fig. 4F). This indicated that the increase in p53 phosphorylation and expression induced by UVB could be suppressed by melatonin. These results showed that melatonin could partly inhibit premature senescence induced by UVB irradiation, along with the inhibition of protein levels and phosphorylation of p53 in primary melanocytes.

### Melatonin partly inhibits senescence-associated pigmentation through the p53-TYR pathway in primary melanocytes after UVB irradiation

**Fig. 5:**
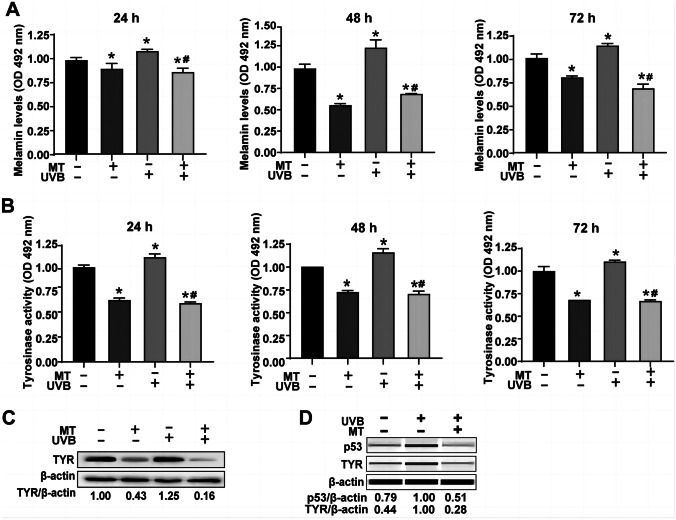
Effects of melatonin on the melanin level, tyrosinase activity, and TYR protein expression in the primary melanocytes after UVB irradiation. **A** and **B** Melanin expression (**A**) and TYR activity (**B**) in the primary melanocytes pretreated with 10^–5^ mol/L of melatonin were measured at 24/48/72 h after UVB irradiation. **C** and **D** The TYR and p53 protein levels in primary melanocytes pretreated with 10^–5^ mol/L of melatonin were analyzed at 72 h after UVB irradiation. (*P < 0.05 vs. the control group; ^#^ *P* < 0.05 vs. the UVB group)

We measured the melanin levels in melatonin-treated or untreated primary melanocytes at 24, 48, and 72 h after UVB irradiation at 80 mJ/cm^2^ to determine the role of melatonin in melanin production in UVB-stimulated primary melanocytes. Melatonin significantly decreased the melanin level (*P* < 0.05) (Fig. 5A). UVB increased melanin expression (*P* < 0.05), which was mitigated in part by pretreated melatonin (*P* < 0.05). Additionally, UVB increased tyrosinase activity (*P* < 0.05). In contrast, the increase was alleviated by pretreated melatonin (*P* < 0.05) (Fig. 5B). The expression of p53 and TYR was assessed in primary melanocytes at 72 h after UVB irradiation to better understand the mechanism contributing to the anti-melanin synthesis effects of melatonin. These results suggested that melatonin decreased the expression of TYR by 57% (Fig. 5C). UVB increased the expression of p53 and TYR, which was significantly reversed by pretreated melatonin (Fig. 5D). Therefore, after UVB irradiation, melatonin can partly inhibit senescence-associated pigmentation through the p53-TYR pathway in the primary melanocytes.

### Melanin synthesis depends on the expression of TYR in hair, whiskers, skin, eyes, and paws in the C57BL/6 J mice

**Fig. 6:**
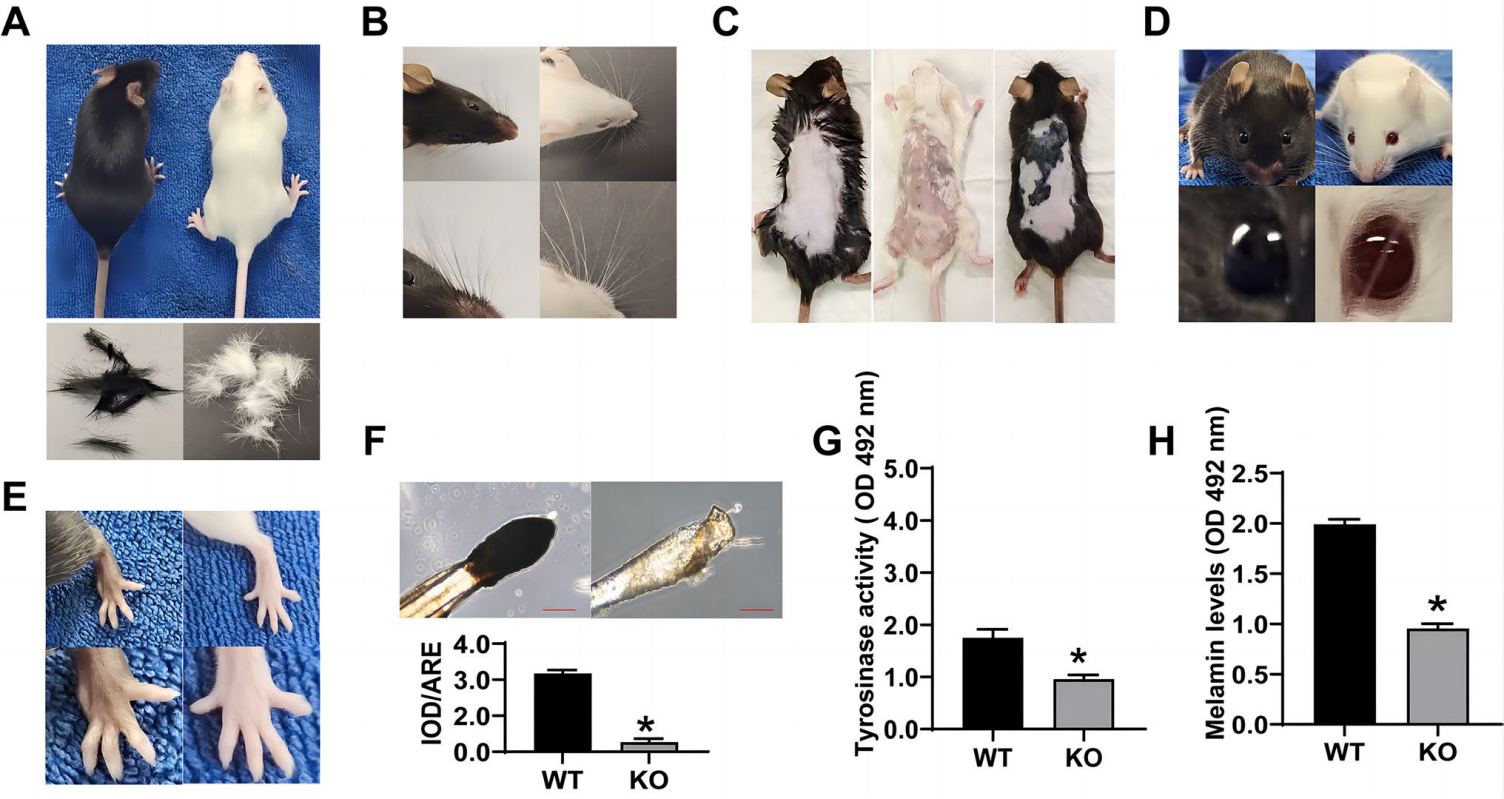
Melanin content in hair, whiskers, skin, eyes, and paws of wild-type and TYR knockout mice. Hair (**A**), whiskers (**B**), eyes (**D**), and paws (**E**) of *TYR*^*(*+*/*+*)*^ (left) and *TYR*^*(–/–)*^ knockout (right) mice were examined 8 weeks after birth. The skin samples (**C**) of *TYR*^*(*+*/*+*)*^ (left), *TYR*^*(–/–)*^ knockout homozygous (middle), and *TYR*^*(–/+)*^ heterozygous (right) mice were examined 8 weeks after birth. Quantification of L-DOPA staining (**F**), tyrosinase activity (**G**), and the melanin levels (**H**) in the whisker hair follicles of the *TYR*^*(*+*/*+*)*^ (left) and the *TYR*^*(–/–)*^ knockout homozygous (right) mice 8 weeks after birth. The *TYR*.^*(*+*/*+*)*^ mice were used as the control. (Scale bar indicates 50 µm. *P < 0.05 vs. the control group)

The *TYR*^*(*+*/*+*)*^ and *TYR*^*(–/–)*^ or *TYR*^*(*+*/–)*^ C57BL/6 J mice showed the effect of TYR on melanin synthesis in vivo. The *TYR*^*(–/–)*^ knockout mice had white coat color, paws (Fig. 6B–E), and hair (Fig. 6A). There was no significant difference in back skin pigmentation in the *TYR*^*(*+*/*+*)*^ (left) mice compared to the *TYR*^*(–/–)*^ knockout homozygous (middle) mice. In contrast, the back skin pigmentation increased in the *TYR*^*(–/*+*)*^ heterozygous (right) mice (Fig. 6C). The images showed a loss of pigment in the eyes of *TYR*^*(–/–)*^ knockout mice (Fig. 6D). Whisker follicles were extracted, stained with DOPA, and captured at a 200 × magnification under the microscope. The follicles of *TYR*^*(*+*/*+*)*^ (left) mice stained deep black, while those of the *TYR*^*(–/–)*^ knockout (right) mice did not (Fig. 6F). The tyrosinase activity and melanin levels in whisker follicles of *TYR*^*(–/–)*^ knockout mice also decreased significantly relative to the WT counterparts (Fig. 6G, H). These results indicated that melanin synthesis in the hair, whiskers, skin, eyes, and paws of the C57BL/6 J mice depended on TYR expression.

### Melatonin prevents skin erythema and melanin pigmentation induced by UVB irradiation in C57BL/6 J mice

**Fig. 7:**
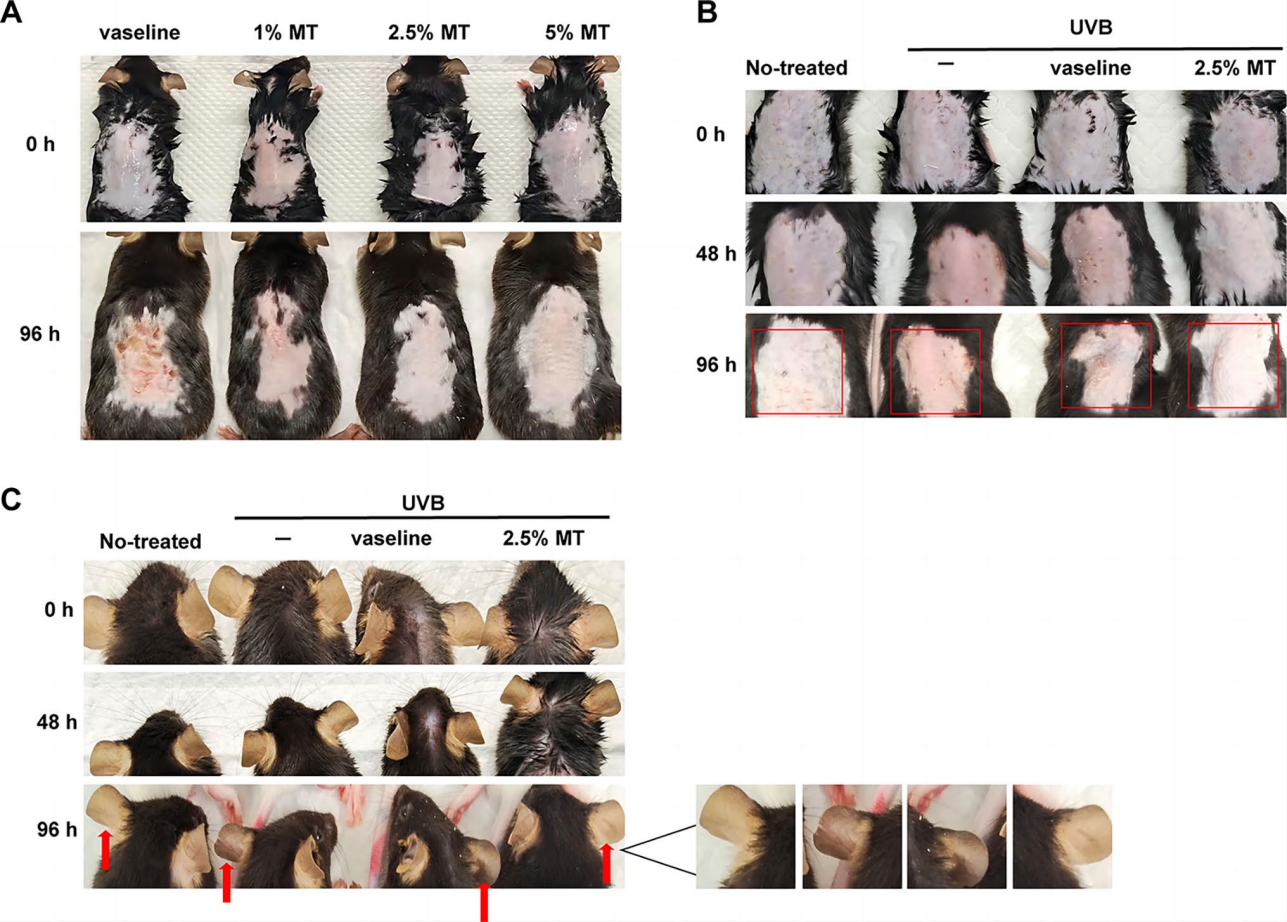
Effect of melatonin on skin erythema and melanin pigmentation induced by UVB irradiation in C57BL/6 J mice. **A** Skin erythema changes in the dorsal skin of mice after treatment with different concentrations of melatonin before irradiation were recorded at 0 and 96 h after UVB irradiation,* n* = 3. **B** and **C** Skin erythema changes in the dorsal skin (**B**) and differences in melanin pigmentation among the ear skin (**C**) of mice in different treatment groups were recorded at 0, 48, and 96 h after UVB irradiation. (Boxes indicate skin erythema changes; arrows indicate melanin pigmentation)

The mechanism by which melatonin affected skin erythema and melanin synthesis after UVB irradiation in C57BL/6 J mice was investigated. First, we discovered that melatonin at a concentration of 2.5% provided the best protection against skin erythema in mice after irradiation (Fig. 7A). Then, we randomized the wild-type *TYR*^*(*+*/*+*)*^ mice into four groups, three individuals per group, to investigate the changes that occurred during skin erythema and melanin pigmentation in mice after UVB irradiation. Moreover, UVB induced excessive damage to the erythema and integrity of the back skin and increased melanin pigmentation in the ear skin of mice (Fig. 7B, C). However, topical treatment with 2.5% melatonin prevented skin erythema and restored its integrity (Fig. 7B). Melanin pigmentation in the ear skin also decreased with topical treatment with 2.5% melatonin in mice after UVB irradiation (Fig. 7C). These results suggested that melatonin prevented skin erythema and melanin pigmentation in C57BL/6 J mice after UVB irradiation.

## Discussion

The skin acts as a barrier that depends on melanocytes to generate melanin for photoprotection and regulates the balance of the human body through local neuroendocrine and immune systems [23, 24]. Melanocytes mainly exist at the junction between the epidermis and dermis, accounting for about 10% of basal epidermal cells, and can synthesize melanin [25]. Primary melanocytes, extracted from the stratum basale of the adult human foreskin, can simulate melanin synthesis in vivo [26]. Primary melanocytes were used as the experimental model, which were identified using various methods. Primary melanocytes have a prominent dendritic structure, a larger cell volume, and higher TYR mRNA and protein levels, which is consistent with previous research findings [27].

Prolonged exposure to UVB facilitates skin senescence and senescence-associated pigmentation [28, 29]. The regulatory mechanisms causing pigmentation are complex and not completely understood. However, several studies have suggested that DNA damage and repair initiate the signaling pathways that increase melanogenesis after UVB irradiation [30]. Our previous study showed that UVB irradiation promoted melanin production in melanocytes, accompanied by tyrosinase activation. Tyrosinase is a rate-limiting enzyme responsible for melanin synthesis [31]. Its activity increases in melanocytes after UVB irradiation [32, 33]. Therefore, TYR gene expression was knocked down using pLKD-CMV-EGFP-2A-Puro-U6-TYR to determine whether UVB-induced melanin synthesis depends on TYR in primary melanocytes. In addition, tyrosinase activity and melanin levels were detected in primary melanocytes at 72 h after UVB irradiation. In this study, we discovered that UVB-induced melanin synthesis partly depends on TYR in the primary melanocytes. In vivo studies with wild-type and TYR knockout C57BL/6 J mice verified the results.

UVB can penetrate the epidermis to induce DNA damage in the melanocytes between the epidermis and dermis [34]. When DNA damage is not repaired properly, melanocytes stop dividing and show symptoms of premature senescence [35]. In our previous study, UVB-induced premature senescence in primary melanocytes was accompanied by an increase in the expression of p53. We determined the critical factors associated with premature senescence and senescence-associated pigmentation and evaluated the effect of premature senescence on pigmentation among primary melanocytes after UVB irradiation. The activator (Nutlin-3) and inhibitor (PFT-α) of p53 were used to upregulate or downregulate p53. Thus, an increase in premature senescence, tyrosinase activity, and melanin levels due to UVB irradiation further increased after Nutlin-3 treatment but decreased significantly after treatment with PFT-α (*P* < 0.05) in primary melanocytes. These findings suggest that activating or inhibiting p53 after UVB irradiation significantly impacts senescence-associated pigmentation by regulating premature senescence. The results were similar to those of previous studies, which suggested the involvement of p53 in paracrine-associated pigmentation and the regulation of the expression of pigment-related genes [36–41]. However, neither TYR nor tyrosinase-related protein-1 (TRP-1) can regulate the p53 gene in humans. The TYR’s expression changes were further assessed after the upregulation or downregulation of p53 in primary melanocytes at 72 h after UVB irradiation. These results showed that p53 regulates TYR expression in primary melanocytes after irradiation. Here, we demonstrated that UVB-induced pigmentation in the skin via TYR regulated by p53, which is consistent with the report of Slominski A that eumelanin production is independent from POMC expression [42].

Melatonin can be used to effectively and safely treat insomnia, anti-oxidation, and anti-aging, with different effects on melanin synthesis among different cell types. It promotes melanin synthesis in the human SK-MEL-1 melanoma cells [43]. It also inhibits melanin synthesis in mammalian hair follicles in vitro, rodent melanoma cells, mouse skin in vitro, and human MNT-1 melanoma cells [44–47]. In this study, we found that melatonin prevented skin damage and melanin pigmentation induced by UVB irradiation in the dorsal and ear skin of C57BL/6 J mice in vivo. To investigate the role of TYR in melanin synthesis, we used the *TYR*^*(–/–)*^ knockout homozygous and *TYR*^*(–/*+*)*^ heterozygous mice models. The use of mouse models still has some limitations, considering that there are some differences in the physiological of skin characteristics between mice and humans. But mice are currently the most appropriate in vivo evaluation model for pigmentation [48]. Previous studies have reported that melatonin and its metabolites inhibit tyrosinase activity in human skin melanocytes, and the local melatonin supplements protect skin from oxidative damage during vitiligo [49, 50]. Our results also showed that melatonin partly inhibited UVB irradiation-induced premature senescence, along with a decrease in p53 phosphorylation and expression levels, and alleviated the increase in tyrosinase activity and melanin levels after UVB irradiation by reducing the expression of TYR. Additionally, p53 can modulate TYR in the primary melanocytes after irradiation. In conclusion, melatonin partly inhibits senescence-associated pigmentation through the p53-TYR pathway in primary melanocytes after UVB irradiation.

## Supplementary Information

Below is the link to the electronic supplementary material.

Supplementary file1 (DOCX 1760 KB)

## Data Availability

The authors declare that all data supporting the findings of this study are available within the article or from the corresponding author upon reasonable request.
