# Supplementary material for: Melatonin inhibits senescence-associated melanin pigmentation through the p53-TYR pathway in human primary melanocytes and the skin of C57BL/6 J mice after UVB irradiation
Source: J Mol Med (Berl). 2023 Apr 10;101(5):581–93. doi: 10.1007/s00109-023-02301-y (PMC10163137; doi:10.1007/s00109-023-02301-y)
Supplement: Supplementary file 1 — Supplementary file1 (DOCX 1760 KB) [file 109_2023_2301_MOESM1_ESM.docx]

**Supplementary Material**

**Melatonin inhibits senescence-associated melanin pigmentation through the p53-TYR pathway in human** **primary melanocytes and the skin of C57BL/6J mice after UVB irradiation**

1. Supplementary materials for Figure 3A in the manuscript.


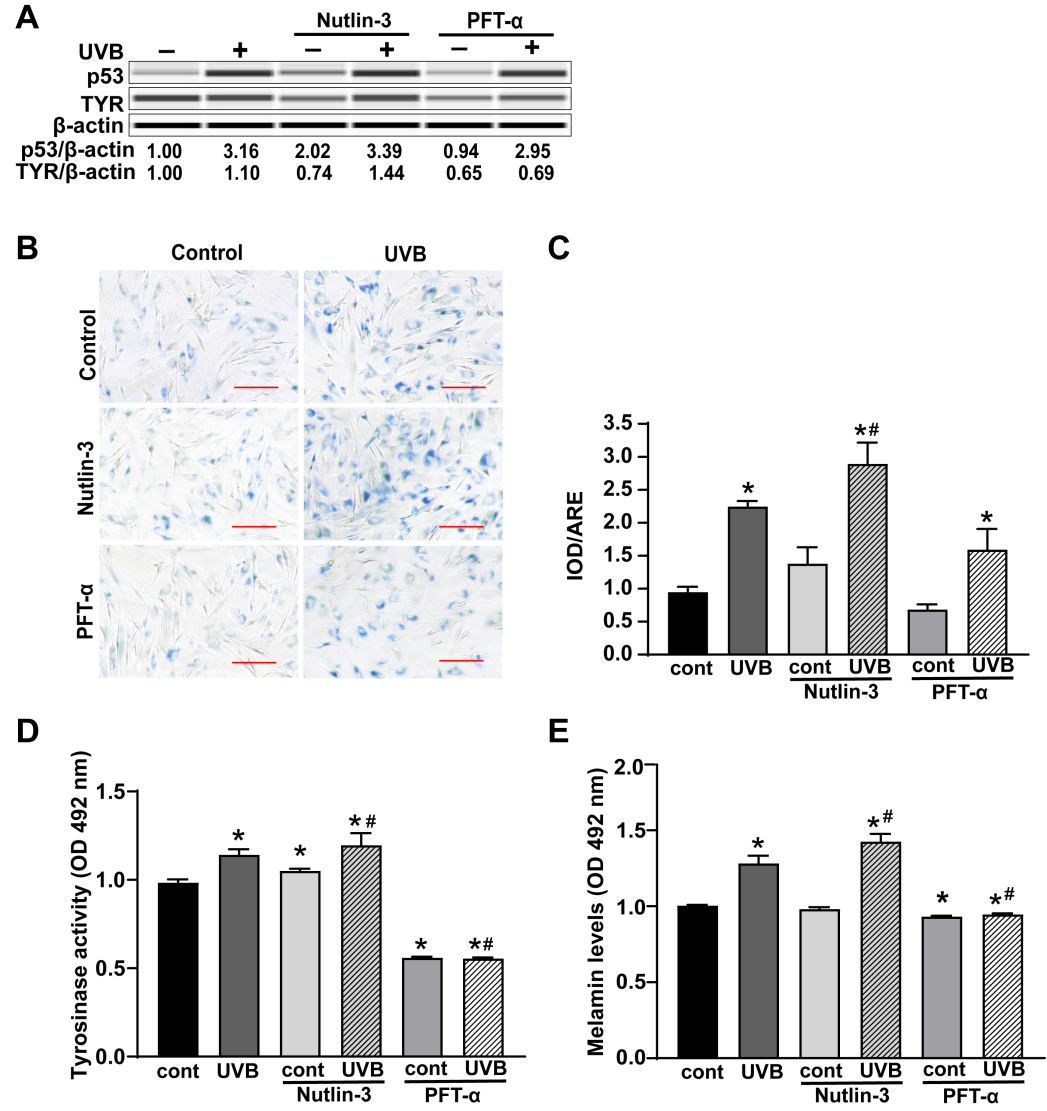


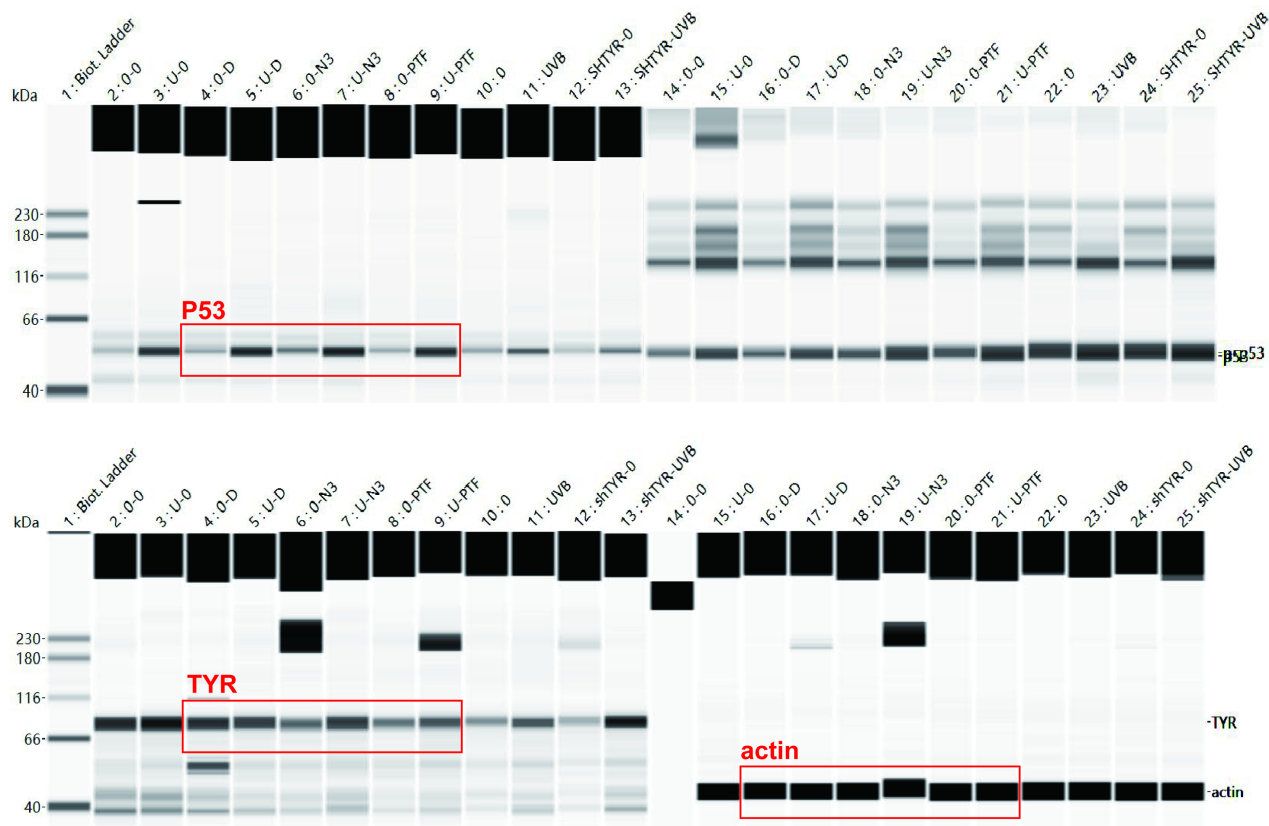


**Supplementary Figure 1.** The supplementary full blots of **Figure 3A** are in the manuscript.

1. Supplementary materials for Figure 4F in the manuscript.


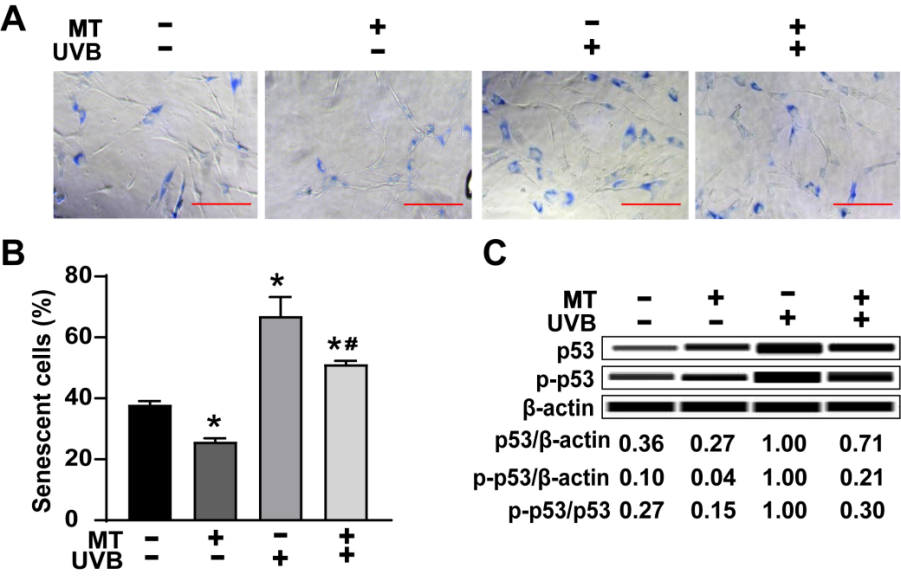


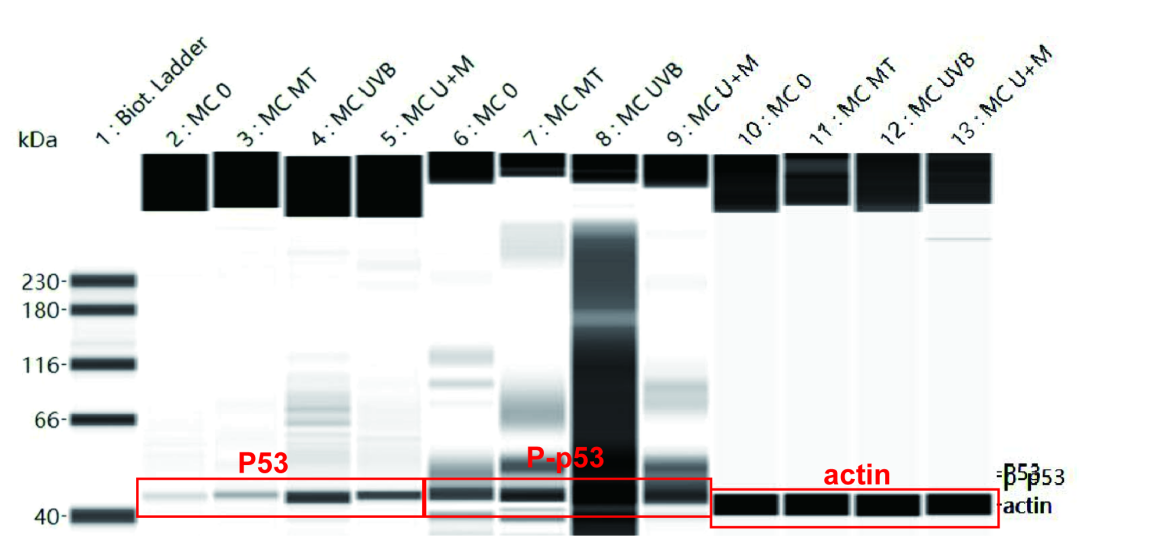


1. Supplementary materials for Figure 5D in the manuscript.


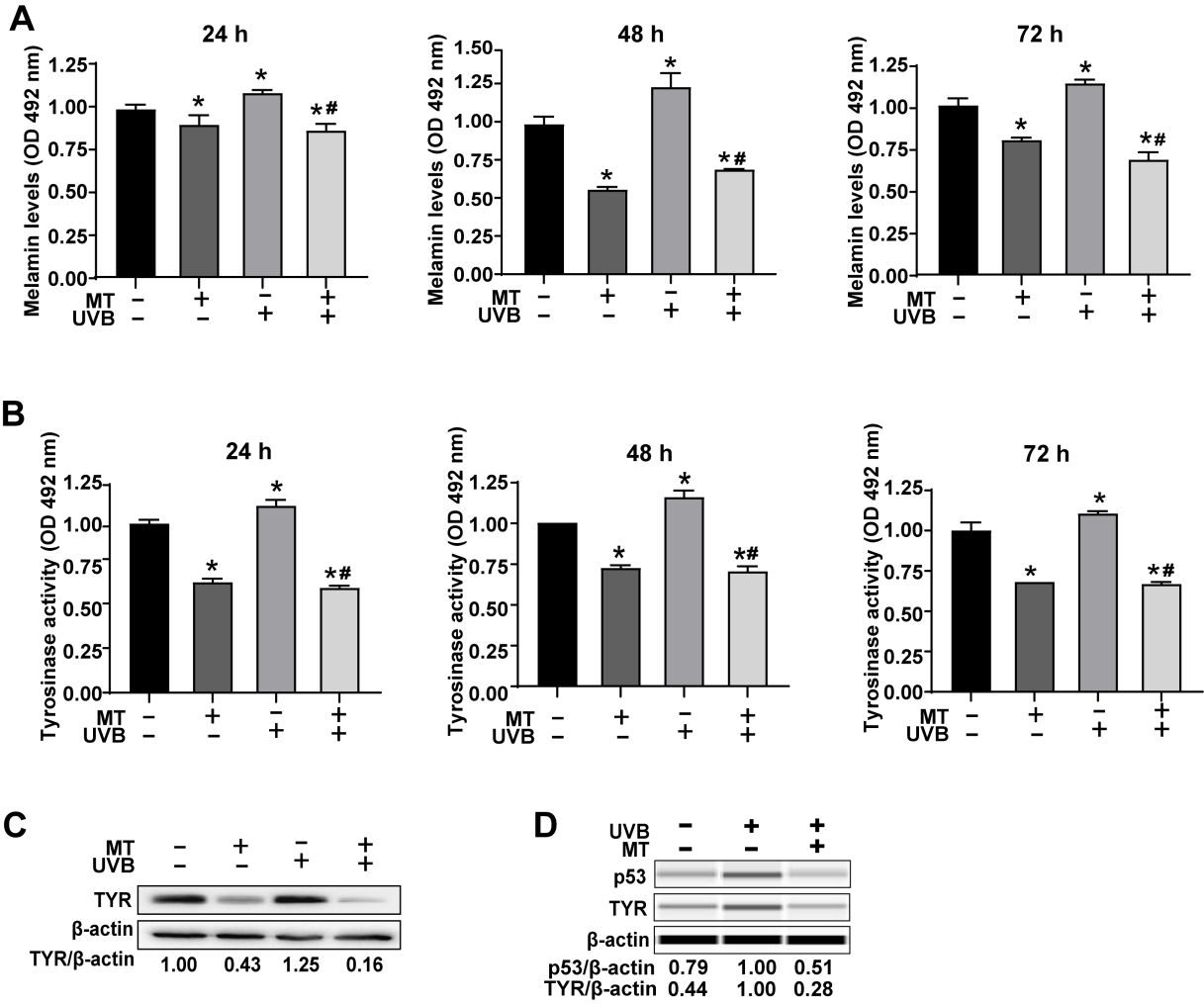
**
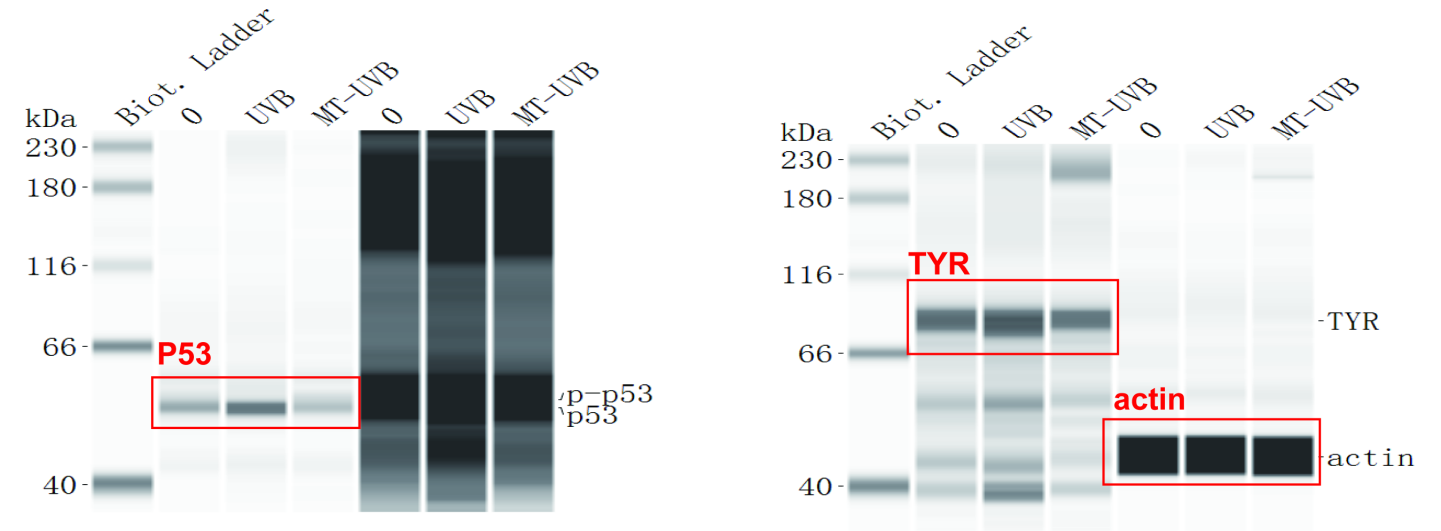
**
